# 2D:4D Digit Ratio Predicts Delay of Gratification in Preschoolers

**DOI:** 10.1371/journal.pone.0114394

**Published:** 2014-12-09

**Authors:** Sergio Da Silva, Bruno Moreira, Newton Da Costa

**Affiliations:** 1 Graduate Program in Economics, Federal University of Santa Catarina, Florianopolis SC, 88049-970, Brazil; 2 Federal Institute of Minas Gerais, Formiga MG, 35570-000, Brazil; University Hospital of Münster, Germany

## Abstract

We replicate the Stanford marshmallow experiment with a sample of 141 preschoolers and find a correlation between lack of self-control and 2D:4D digit ratio. Children with low 2D:4D digit ratio are less likely to delay gratification. Low 2D:4D digit ratio may indicate high fetal testosterone. If this hypothesis is true, our finding means high fetal testosterone children are less likely to delay gratification.

## Introduction

In a famous experiment conducted 40 years ago at Stanford [1], when a group of 4 year olds were faced with a choice between a small reward (one Oreo cookie), which they could have at any time, or a larger reward (two cookies), for which they had to wait 15 minutes, about half the children managed to delay gratification. As young adults, the children who had demonstrated greater self-control had higher scores on intelligence tests and were less likely to take drugs. Executive attention, or the ability to regulate responses, is known to develop under strong genetic control although it is also amenable to educational training during development [2]. Resistance to temptation as measured by the cookie test is a lifelong individual difference that predicts reliable biases in frontostriatal circuitries, which integrate motivational and control processes [3].

The Stanford experiment was replicated many times using other rewards, including marshmallows. Here, we revisit the experiment to further consider: children's gender, 2D:4D digit ratio and handedness. The digit ratio indicates the relative lengths of the second (“index”) finger and the fourth (“ring”) finger, and may be a biomarker of fetal testosterone levels [4].

The balance between fetal testosterone and fetal estrogen influences 2D:4D formation and a low 2D:4D ratio indicates high fetal testosterone and low fetal estrogen [5]. It is accepted [6] that adult males tend to have lower average 2D:4D ratios (∼0.98) than adult females (∼1). With regard to the ratio among children, a previous study (which two of the authors of this study contributed to [7]) found that male preschoolers had an average 2D:4D ratio of 0.973 while their female peers had an average ratio of 0.989. Children therefore tend to have similar ratios to those of adults. As prenatal androgens masculinize the human central nervous system [8], the prospect that 2D:4D represents a retrospective marker of the level of androgen exposure during the period when the brain is sexually differentiated has fueled vast literature [9], [10], which grows exponentially [5].

The key support for the hypothesis that 2D:4D ratio reflects fetal testosterone is that the magnitude of 2D:4D covaries with a polymorphic repeat (number of polyglutamine CAG) sequence in exon 1 of the gene that encodes the androgen receptor [11]. This finding has been challenged recently [12], [13]. However, rather than the more inexact correlational evidence, the hypothesis still receives support from evidence that involves experimental manipulation of 2D:4D in rodents [14], [15] and the use of exogenous testosterone in humans [16].

## Materials and Methods

**Figure 1:**
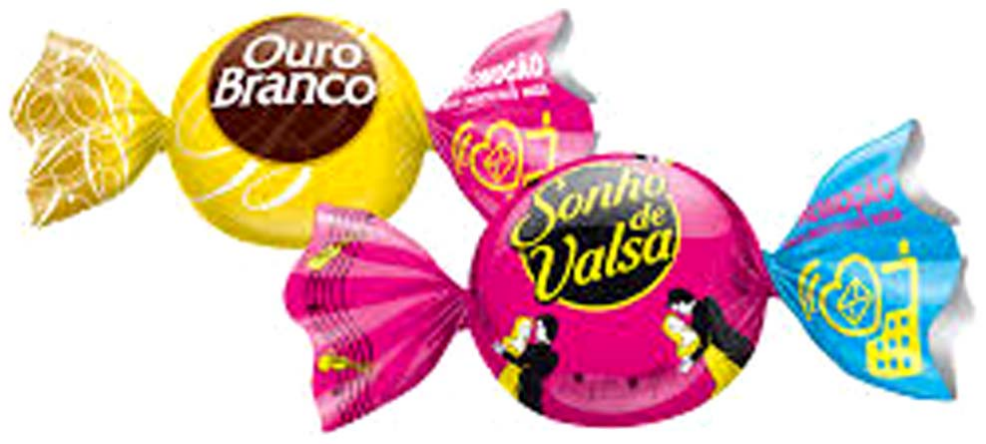
Candies used in the experiment.

We ran field experiments inspired by the classic marshmallow test. The experiments took place at six kindergartens (five high-income schools and one lower-income school) from September to December 2012 in Florianopolis, a city in southern Brazil. A total of 141 children (67 males and 74 females), ages 4 to 6 took part (data available at http://dx.doi.org/10.6084/m9.figshare.1160513). Children between 4 and 6 already consider themselves to be autonomous individuals, capable of counting and understanding quantities, and able to realize that events may have a cause. The research was approved by the Ethical Committee at the Federal University of Santa Catarina (approval number: 057/08; approval date: April 10, 2008; decision published: April 28, 2008). Parents were asked to allow their children to participate before providing consent in writing. Rather than offering the children marshmallows or Oreo cookies, we used candies that are popular in Brazil, as shown in Fig. 1.

Each child was given one candy at the beginning of class. The experimenter (B.M.) then explained to the children that they had to wait for the teacher's permission before eating the candy. Next, the teacher told the children they could eat the candy at any time during class. Additionally, the teacher also explained that, if they resisted the temptation to eat the first candy and waited for further instructions, then they would be given another candy as a reward. After 20 minutes, the teacher offered a second candy to those children who had resisted temptation and refrained from eating the first candy.

In contrast to the classic experiment by Mischel et al. [1], the children were not tested alone, but were seated next to each other at small tables. This is by no means a drawback of our experiment. On the contrary, this means that our test is actually more stringent than the classic one, because self-control is more difficult when children imitate peers. Indeed, in a previous study, Nisan [17] offered children a choice between an immediate food reward and a delayed, but larger reward. The children made the decision either alone or in a small, same-sex peer group. Choices made by girls did not differ, irrespective of whether deciding alone or in groups, whereas when the boys were making decisions in groups, the first choice that was suggested prevailed as the final decision. More recently, McCabe and Brooks-Gunn [18] found that children are less able to demonstrate self-control in peer groups, compared to when they are assessed individually. Children in groups tended to imitate the behavior of those around them, and were less capable of exerting self-control than children tested alone.

The 2D:4D ratio was measured by the experimenter after tracing the contour of each child's right hand on a sheet of white paper. This is far from ideal and was the result of a compromise with schools' principals. Measuring with a caliper (probably the most accurate method) was seen as time consuming, and most principals tended to view the caliper as too intrusive, which added little in terms of pedagogical value. Even if calipers were allowed, we presume we would experience difficulty in measuring, due to the short attention span of the children. Furthermore, photocopying or scanning young children's hands would likely result in many blurred and unusable images. In the end, tracing around the fingers is probably unacceptable for adult participants, but may represent a best-practice solution for 4 to 6 year olds. Indeed, the children seemed very relaxed during the task, reflecting the playful aspect of the chosen measuring technique. The experimenter also noted cooperation on behalf of the children. This may be related to the fact that this was not the first time they engaged in the task of tracing the contour of their hands, that is, this activity was previously introduced by the teachers. The finger lengths measured from the tracings showed roughly the same results across the six kindergartens. More importantly, there was repetition in the measurements with regard to a previous measurement of an earlier study we conducted in the same six kindergarten classes [19]. In short, the measurement seemed robust across kindergarten classes and across time. Children's anonymity was preserved, and only their gender was recorded by the experimenter.

We also noted the children's handedness. Many left-handed people have been reported to have IQs greater than 140 compared to right-handed people, and are associated with musical talent and athleticism. This may be partly because left-handed people have an intrinsic neurological advantage over right-handed people [20]. Because gratification delay in children can be a predictor of higher scores on intelligence tests later in life, we hypothesized that left-handedness might be linked to the ability to delay gratification. However, this hypothesis failed to be validated. The experimenter first asked teachers to report the handedness of the participants and then confirmed their answers by asking a child to make a drawing on a sheet of paper. Using the hands for drawing has been argued to be the best way to assess child's hand preference [21]. In our sample, 105 children were right-handed (62 females and 43 males) and 36 were left-handed (12 females and 24 males). This result of 75.5 percent of right-handed children matches the one in reference [21], where 73 percent were right-handed (in which the children's hand preference was also verified through drawing).

We estimate the model parameters and the influence of candidate explanatory variables (gender, digit ratio and handedness) on the probability of delaying gratification as follows:
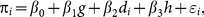
(1)where *π_i_* is the probability of failing to delay gratification for child *i*; *β_j_* is the coefficient vector capturing the impact of changes in the explanatory variables on *π_i_*; *g* is a dummy for gender (female  = 0; male  = 1); *d_i_* is the 2D:4D digit ratio for child *i*; *h* is a dummy for handedness (left- handed  = 0; right-handed  = 1); and ε*_i_* is an error term.

An explanatory variable selection was conducted using stepwise, backward and forward regressions. It was found that neither gender or handedness were significant for explaining *π_i_*.

## Results

The average 2D:4D ratio for the sample was 0.963. As expected, the average ratio for male children was smaller (mean  = 0.943; SD  = 0.027) than for females (mean  = 0.981; SD  = 0.014), and the *t* test regarding mean differences in male and female 2D:4D showed a *p*-value <0.001. This result is in line with our previous finding [7] and it may reflect that boys have higher fetal testosterone than girls.

We found that 105 out of 141 children failed to delay gratification, which is a massive 74.47 percent. These findings replicate those of the Stanford study [1]. Moreover, we found that the 2D:4D ratio was associated with failure to delay gratification (*p*<0.017; *z* = −2.38). The estimated logistic equation (2) shows the significant result after consideration of gender, digit ratio and handedness as candidate explanatory variables:
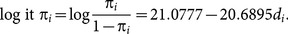
(2)


Taking the logs on both sides, equation (2) becomes:
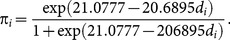
(3)


**Figure 2:**
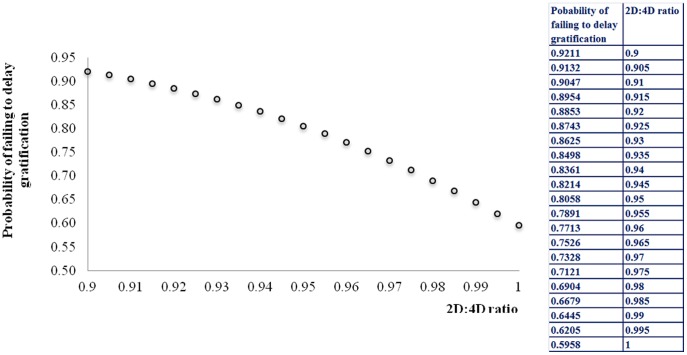
Failure to delay gratification is more likely the lower the digit ratio.

Considering the 2D:4D ratio measures found for the children in equation (3), one can find the associated probability of failing to delay gratification (Fig. 2). Fig. 2 illustrates an inverse relationship between *π_i_* and *d_i_*. For instance, children with 2D:4D = 0.9 have a 92.11 percent chance of failing to delay gratification. In contrast, children exhibiting 2D:4D = 1 (quite possibly females) have only a 59.58 percent chance of failing to delay gratification. Therefore, failure to delay gratification is more likely the lower the digit ratio.

A low 2D:4D digit ratio may indicate high fetal testosterone, as observed. If this hypothesis is true, our finding means high fetal testosterone children are less likely to delay gratification. The link between 2D:4D and fetal testosterone may make sense because of evidence that relates salivary testosterone and delay-discounting behavior. The quality of frontostriatal white matter tracts can predict individual differences in delay-discounting behavior [22]. The authors in reference [22] consider tract-based diffusion tensor imaging and magnetization transfer imaging to measure the microstructural properties of frontostriatal fiber tracts in 40 healthy young adults (ages 18 to 25). Additionally, they explored whether internal sex hormone levels affect the integrity of frontostriatal tracts. In one measure (radial diffusivity), testosterone levels in males were associated with a lower integrity within the frontostriatal tract.

## Conclusions

This study considered the relationship between the probability of delay of gratification (a delay in eating candy to receive more candy) and three variables: gender, 2D:4D digit ratio and handedness. The participants were 141 preschool children (67 boys) ages 4 to 6. We found 105 (74.5 percent) participants failed to delay gratification, and that failure to delay gratification was dependent on 2D:4D, but not on gender or handedness. Children with a lower 2D:4D ratio (possibly corresponding to higher fetal testosterone) failed to delay gratification more often than those with a higher digit ratio. Therefore, the results posit an inverse relationship between a delay of gratification in preschoolers and their digit ratios.
